# Transcranial Alternating Current Stimulation as an Adjuvant for Nonfluent Aphasia: A Proof-of-Concept Study

**DOI:** 10.3390/bioengineering13030372

**Published:** 2026-03-23

**Authors:** Lynsey M. Keator, Lisa Johnson, Roger Newman-Norlund, Kyler Spell, Samaneh Nemati, Leigh Ann Spell, Dirk B. den Ouden, Christopher Rorden, Julius Fridriksson

**Affiliations:** 1 Department of Speech-Language Pathology, College of Rehabilitation Sciences, Thomas Jefferson University, 130 South 9th Street, Philadelphia, PA 19107, USA; 2 Department of Communication Sciences and Disorders, University of South Carolina, 915 Green Street, Columbia, SC 29201, USA; lajohnson.usc@gmail.com (L.J.); kylerpspell@gmail.com (K.S.); snemati@mailbox.sc.edu (S.N.); fridriks@mailbox.sc.edu (J.F.); 3 Department of Psychology, University of South Carolina, 915 Green Street, Columbia, SC 29201, USA; rnorlund@mailbox.sc.edu (R.N.-N.); rorden@mailbox.sc.edu (C.R.); 4 Speech-Language Pathology Program, School of Health Sciences, Columbia College, 1301 Columbia College Drive, Columbia, SC 29203, USA; lspell@columbiasc.edu; 5 Communication Sciences and Disorders, Crean College of Health and Behavioral Sciences, Chapman University, 9401 Jeronimo Road, Irvine, CA 92618, USA; denouden@chapman.edu

**Keywords:** transcranial alternating current stimulation (tACS), nonfluent aphasia, speech-language therapy, noninvasive brain stimulation

## Abstract

Effective rehabilitation tools are essential for improving language outcomes in chronic aphasia. Speech entrainment is a behavioral treatment that has shown promise in enhancing speech output in nonfluent aphasia, potentially by acting as an external mechanism to synchronize anterior and posterior language regions in the left hemisphere. Transcranial alternating current stimulation has been hypothesized to enhance functional connectivity between brain regions by amplifying endogenous oscillations. This proof-of-concept study explored whether high-definition tACS (HD-tACS) could improve speech fluency in nonfluent aphasia when paired with speech entrainment. In a double-blind, pseudorandomized study, 1 mA of HD-tACS at 7 Hz was applied to anterior and posterior left-hemisphere regions of individuals with nonfluent aphasia (N = 13). Stimulation was applied under three conditions: in-phase, anti-phase, and sham, and paired speech entrainment. Three outcome measures were examined: (1) number of words produced; (2) number of errors, and (3) ‘entrainment’ to the speech entrainment model. Group-level analyses for two of the three outcome measures reveal statistically significant differences between the experimental conditions. In-phase alternating current stimulation yielded more words and better entrainment to the audiovisual model than the sham condition. This study provides promising evidence that HD-tACS could improve speech production in individuals with nonfluent aphasia. These results contribute to growing evidence supporting the therapeutic potential of non-invasive brain stimulation approaches as an adjuvant to traditional behavioral speech-language therapy in stroke survivors.

## 1. Introduction

Stroke is a leading cause of mortality and disability, affecting millions globally each year. One of the most devastating consequences of stroke is aphasia, a language disorder resulting from damage to the brain’s language centers, typically in the left hemisphere. Approximately 20 to 30 percent of stroke survivors experience aphasia [1,2], with many enduring its effects for years into the chronic stages of recovery [1,3,4]. Aphasia severely impairs communication, leading to significant social isolation, emotional distress, and a profound reduction in quality of life [5]. Given the high prevalence and debilitating impact of aphasia, effective rehabilitation tools are essential for improving language outcomes.

Nonfluent aphasia is a subtype of aphasia characterized by impairments in speech production, including reduced verbal output, slow and effortful speech, frequent pauses, and poor articulation [4,6,7,8]. Nonfluent aphasia affects approximately 40% of individuals with chronic aphasia [9,10,11]. Contrary to historical accounts, contemporary research suggests that lesions responsible for nonfluent aphasia may not be isolated solely to anterior regions of the left hemisphere. For example, nonfluent aphasia may result from damage that is pervasive across anterior and posterior (superior temporal gyrus [STG] and inferior parietal lobe) cortical structures in the left hemisphere [12]. From a theoretical perspective, it is hypothesized that the nonfluent speech characteristic of this aphasia subtype is caused by impaired motor planning and a degraded or absent efferent copy, at least in a subset of these patients [13,14,15]. The efference copy is a feed-forward projection of the motor plan that is recruited to predict sensory feedback. In the domain of speech and language, the efference copy is thought to be an internal representation of motor speech plans that predict speech behaviors and facilitate fluent speech production [13,16,17]. It is hypothesized that the left-lateralized dorsal stream [18,19] integrates sensorimotor networks to monitor auditory feedback online by comparing predicted and actual inputs. Therefore, in the context of nonfluent aphasia, which is caused by damage to left hemisphere language regions that house feedforward projections (i.e., expected speech output), the left hemisphere damage prohibits the generation of an efference copy, thereby hindering the initiation of the speech production mechanism [13,14]. Efficient coupling between frontal and temporal cortices is thought to initiate a comparison between the intended speech (efference copy) and actual speech output [20]. Given the underlying neural correlates of the proposed efference copy, it seems reasonable to suggest that a therapeutic intervention that acts as an external gaiting mechanism to initiate and monitor the flow of speech in the presence of a damaged efference copy may improve speech fluency [14,15].

Behavioral interventions, such as speech-language therapy, are the primary treatments for nonfluent aphasia. These therapies aim to improve speech fluency through verbal repetition and visual stimuli, but often result in errors and limited practice due to the challenges of eliciting speech [21,22]. Consequently, many patients with nonfluent aphasia experience minimal improvement and persistent fluency impairments. One promising rehabilitation approach is speech entrainment (SE), which involves synchronizing speech production with an external audiovisual model. Unlike traditional therapeutic approaches that prompt patients to generate and produce speech, SE yields promising improvements in speech fluency by guiding or pulling along’ the patient’s speech [14,15].

Speech entrainment relies on action observation and real-time rehearsal of situation-specific scripts. This technique has been shown to enhance speech output, particularly in individuals with nonfluent aphasia [14,15]. One reason SE has proved successful is the audiovisual stimulus, which improves speech production in patients with nonfluent aphasia compared with audio-only stimuli [23]. Audiovisual stimuli have also been associated with distinct patterns of cortical activation. In a cohort of healthy controls, stronger activation was observed in the inferior frontal gyrus pars opercularis (IFGpo) and the posterior middle temporal gyrus (pMTG) during visual and audiovisual tasks compared to audio-only speech conditions [24]. Successful entrainment in patients with nonfluent aphasia has been associated with the integrity of left-hemisphere ventral stream regions, particularly the left pMTG [14,25], given the role of the left pMTG in audiovisual integration [24]. Furthermore, investigations of the functional connectivity underlying successful entrainment suggest that persons with aphasia demonstrate increased functional connectivity during speech entrainment compared to free speech tasks across anterior (IFGpo) and posterior (pMTG) regions of interest [26]. It has been hypothesized that speech entrainment therapy acts as an external ‘gating’ mechanism or efference copy to support anterior damage and reinforce anterior–posterior coherence in the left hemisphere by regulating the flow of neural signals related to speech production [14,15]. This mechanism may help the speaker compensate for an impaired ability to predict and monitor their own speech-related movements. Speech entrainment therapy may improve synchrony between the anterior and posterior language regions, helping these areas work together more effectively [14,15,27,28].

Emerging research highlights the potential for combining behavioral therapies with innovative biological techniques to significantly improve rehabilitation outcomes [29,30,31,32,33]. Non-invasive brain stimulation techniques, such as transcranial direct current stimulation (tDCS), are effective adjuvants to traditional therapy [34]. There is a growing body of research suggesting that, when paired with behavioral speech-language therapies, non-invasive stimulation can ‘boost’ rehabilitation outcomes and augment synaptic plasticity, thereby inducing functionally relevant changes in the networks that support language [35]. Non-invasive brain stimulation techniques such as repetitive transcranial magnetic stimulation (rTMS) and tDCS are thought to facilitate activity in residual language regions or suppress dysfunctional neuronal processes [36].

Transcranial alternating current stimulation (tACS) has been studied far less than other stimulation approaches in patients with post-stroke aphasia. Unlike tDCS, which delivers constant currents, tACS delivers low, periodically alternating currents to the scalp, modulating neural oscillations and influencing cognitive functions [37,38,39,40]. tACS modulates neural networks by improving coherence between regions rather than overall activity levels [41,42]. Another advantage of tACS is that its stimulation frequency can modulate task-relevant physiological processes [43,44]. tACS is thought to directly facilitate neuronal excitability [44] and to induce rhythmic changes (neural oscillations) at a frequency that corresponds to the stimulation frequency [44,45,46,47,48]. It has been suggested that tACS has the potential to improve neural synchrony in deviant oscillatory function secondary to stroke and, consequently, enhance behavioral outcomes [49,50]. Use of high-definition tACS (HD-tACS) optimizes stimulation and network effects by more precisely constraining stimulation effects [41,45,51,52]. Research from clinical and healthy populations suggests that in-phase tACS, which enhances synchronization and coordination between brain regions, can improve behavioral performance [45,53,54,55], whereas anti-phase tACS interferes with or desynchronizes brain regions, which may impede network synchronization and worsen performance [44]. A limited but growing body of recent research is exploring the effects of tACS as an adjuvant to behavioral language therapy in persons with aphasia [56,57].

One advantage of tACS is that the stimulation frequency can modulate task-relevant physiological processes. In the current study, stimulation was delivered at 7 Hz. This frequency is within the theta frequency band. Neuroanatomically, low-frequency oscillations, such as those in the theta band, are thought to play a primary role in long-range connectivity between anterior and posterior regions [44,58,59,60,61,62,63]. Phase synchronization in the theta band and increased theta coherence between prefrontal and temporal areas are associated with improved language and memory functions [64,65,66,67,68]. In multimodal sensory processing, neural oscillations in different frequency bands reflect distinct aspects of processing [69]. Audiovisual speech, for example, is one type of multimodal sensory processing that aids speech understanding by orchestrating neural oscillations [69,70]. Theta frequency bands are strongly associated with the rhythmic patterns of human speech [71] and are active in multisensory integration (i.e., audiovisual speech). Previous work suggests oscillations are influenced by visual and auditory components of speech because the multisensory stimuli reset the phase of low-frequency oscillations in the auditory cortex [72,73,74,75,76,77] and enhance audiovisual speech processing. Theta-band frequency oscillations were targeted in the current study because of their role in audiovisual speech processing.

This study investigated whether applying HD-tACS at 7 Hz over anterior and posterior left-hemisphere language regions can improve language production and speech timing during a speech entrainment task in individuals with nonfluent aphasia.

## 2. Materials and Methods

### 2.1. Participant Recruitment and Clinical Characteristics

Thirty participants were recruited from past studies at the Aphasia Laboratory, University of South Carolina, based on previously collected behavioral data and additional outreach through a quarterly laboratory newsletter. Recruitment was based on the following inclusion criteria: chronic (>6 months post-onset) nonfluent post-stroke aphasia (Broca’s or transcortical motor) from a single left-hemisphere ischemic stroke, confirmed by magnetic resonance imaging (MRI) or computed tomography (CT). The primary scores used for enrollment were: Western Aphasia Battery-Revised (WAB-R) Aphasia Quotient < 93.8, Fluency subtest < 5, Naming and Word Finding subtest < 9, and Auditory Verbal Comprehension subtest > 4, consistent with the nonfluent aphasia classification on the WAB-R [78]. All participants had completed an MRI at the McCausland Center (Siemens 3-Tesla Prisma Fit Magnetic Resonance Imaging system, 20-channel head coil, Siemens Medical Systems, Erlangen, Germany, Prisma Health Richland (Columbia, SC, USA), and had fMRI data for the Naming 40 task, which involves naming pictures of high-frequency nouns [79]. Participants, aged 30 to 85, were monolingual native English speakers who consented verbally or in writing and passed an initial screening. Exclusion criteria included global aphasia, self-reported dementia, brain injury (excluding stroke), psychiatric disorders, alcohol abuse, or any contraindication for transcranial electrical stimulation (e.g., implanted electronic devices, metal implants, skin sensitivity).

### 2.2. Study Procedures

**Figure 1:**
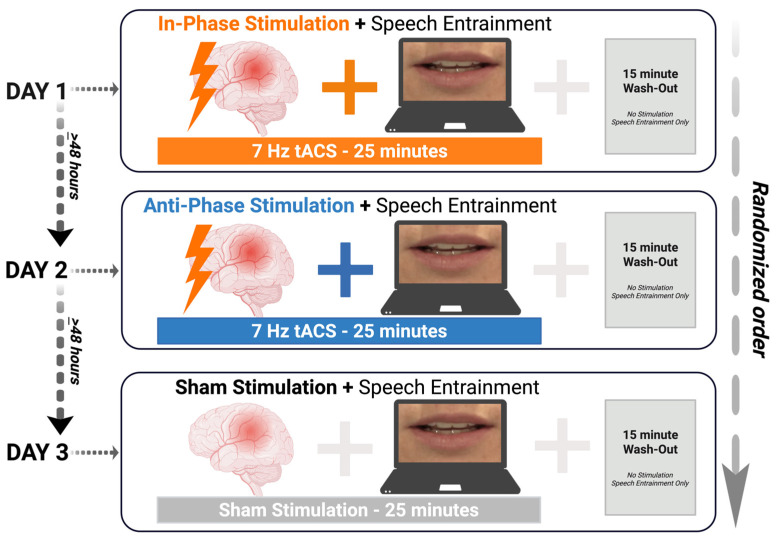
Experimental design. Participants were recruited for three days of a paired non-invasive brain stimulation and behavioral paradigm to participate in three conditions: (1) in-phase stimulation; (2) anti-phase stimulation; and (3) no stimulation (sham). tACS = transcranial alternating current stimulation; SE = speech entrainment; min = minutes. Created in BioRender. Keator, L. (2026) https://BioRender.com/qsvnbul.

Participants were enrolled in a within-subjects, sham-controlled trial with a pseudorandomized block design. Over three days, each visit was separated by at least 48 h. Participants underwent three trials of an HD-tACS + speech entrainment (SE) paradigm under the following conditions: (1) theta-tuned (7 Hz) HD-tACS in-phase montage, (2) theta-tuned (7 Hz) HD-tACS anti-phase montage, and (3) HD-tACS sham condition. HD-tACS (1 mA at 7 Hz) targeted intact anterior (inferior frontal gyrus; pars opercularis; IFGpo) and posterior (posterior middle temporal gyrus; pMTG) regions in the left hemisphere. HD-tACS was applied at 7 Hz, a frequency band involved in human speech-syllable processing [80] and associated with neural oscillatory patterns for audiovisual processing [69,70]. It was hypothesized that an exogenous boost of in-phase theta coupling via HD-tACS would enhance frontotemporal network connectivity and facilitate neural integration across intact anterior and posterior regions, thereby improving speech entrainment performance. The study was double-blinded, with pseudorandomized scripts and stimulation conditions. Figure 1 illustrates the experimental design.

A study administrator and an undergraduate research assistant assigned stimulation conditions and prepared the high-definition transcranial electrical stimulation Soterix system. Speech-language pathology graduate student scorers were blinded to behavioral data, which was coded by color to obscure the order of collection. Data files were anonymized with participant numbers, script labels, and color codes. The study investigator remained blinded to data until all analyses were complete. Data were unblinded only after final analyses.

Data were collected in the Aphasia Laboratory across three sessions: approximately 2 h for the first visit and 1 h for each subsequent visit. The first visit included consent review, administration of the WAB-R, baseline discourse data collection, and a practice session for the speech entrainment paradigm. Each session involved the same speech entrainment task across three conditions: in-phase stimulation, anti-phase stimulation, and sham, with the order pseudorandomized. At the end of each session, participants completed a safety questionnaire [81] and a visual analog scale [82] to assess pain (Wong-Baker FACES Pain Rating Scale [83]), discomfort, and sensory disturbances. Electrode impedance was recorded before and after stimulation, and scales were adapted for participants with aphasia using visual and written aids. After each session, participants and the speech-language pathologist (SLP) guessed the stimulation condition, with cues provided to ensure comprehension.

54 behavioral sessions were conducted over an eight-month period. Due to attrition (n = 1) and reduced verbal output (n = 4), data from 13 participants are included, yielding a total of 39 sessions in the analysis. All participants tolerated tACS well, with no adverse effects reported. Technical errors occurred in 2 of the 54 sessions (4%) due to software updates, resulting in data recording interruptions. Affected participants (T6 and T14) were asked to return for an additional session under identical stimulation conditions and speech entrainment scripts.

### 2.3. Western-Aphasia Battery-Revised Administration

On the first day of data collection, the WAB-R [78] was administered by a speech-language pathologist. The WAB-R Aphasia Quotient (WAB-R AQ), a global measure of aphasia severity (0–100, where a score below 93.8 indicates aphasia), was calculated using scores from the *Spontaneous Speech*, *Auditory Verbal Comprehension*, *Repetition*, and *Naming and Word Finding* subtests. These scores confirmed the presence and severity of nonfluent aphasia and provided a discourse sample (picture description). The WAB-R AQ scale is: 0–25 for very severe aphasia, 26–50 for severe, 51–75 for moderate, and >76 for mild aphasia [78]. The picture description task, part of the *Spontaneous Speech* subtest, was used to assess participants’ spontaneous speech [84].

### 2.4. HD-tACS Administration 

HD-tACS was administered using a Soterix Medical MxN High Definition—Transcranial Stimulation (HD-tES) 9002A system with 9 high-definition electrodes and associated equipment (Soterix Medical, Inc., New York, NY, USA). The system used 12 mm diameter Ag/AgCl electrodes in a high-definition BrainCap, powered by four 10,000 mA h rechargeable batteries (Tenergy Corporation, Fremont, CA, USA). HD-Gel™ (Soterix Medical, Inc., New York, NY, USA) was applied to enhance conductivity. Channels 1–7 were used for stimulation, with channel 8 as the reference electrode. Impedance was checked before and after each session to ensure impedance remained <10 kΩ.

Individualized electrode montages were determined a priori for each participant to target the left inferior frontal gyrus pars opercularis (IFGpo) and left posterior middle temporal gyrus (pMTG), with placements guided by current-flow modeling software (Soterix Medical; HD-Explore^TM^ (Version 6.0.0) and HD-Targets^TM^ (Version 3.0) software; Soterix Medical, New York, NY) and previous fMRI data. Specifically, fMRI data for the Naming 40 task [79] were used. The Naming 40 is a behavioral task that consists of naming pictures of high-frequency common nouns. For additional details on this paradigm, please refer to earlier publications that used the same methodology [85,86,87]. Anterior and posterior stimulation sites were determined by identifying peak activation in the Naming 40 task. An in-house MATLAB (R2021b) script was used to determine coordinates for anterior and posterior stimulation sites which were calculated from an ROI mask for each region (anterior: IFGpo; posterior: pMTG) derived from the Johns Hopkins atlas [88], and peak activation within the ROI mask, but outside of the lesioned area, was used to develop individualized montages.

**Figure 2:**
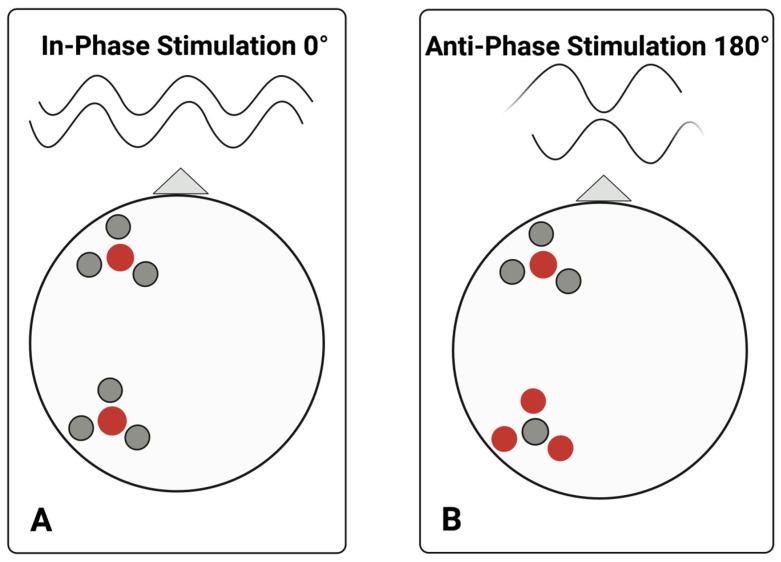
Sample electrode ring montage. This figure illustrates electrode placement and stimulation conditions for in-phase versus anti-phase stimulation. During in-phase or positive cycles of alternating current stimulation ((**A**), i.e., 0° offset), the central electrode in each region of interest continuously carried a current of the same polarity. During anti-phase, or negative cycles of stimulation, HD-tACS is applied at the same location, but the central electrodes share a current of opposite polarity ((**B**), i.e., 180°) across the two targeted cortical regions of the left hemisphere: anterior and posterior each with a central electrode and three surrounding ring electrodes, with respect to the nasion, which is visualized here with a triangle. Red and grey dots indicate current of polarity. A center-surround, course-sink pattern was used to achieve maximum focality. Created in BioRender. Keator, L. (2026) https://BioRender.com/dajxj0q.

Montages were maximized for focality using a ring setup with eight electrodes, ensuring focal stimulation of residual cortex for each participant (consistent with methods from [55]). In each set, maximal stimulation was provided by the center electrode, which overlapped the region of interest, with three surrounding electrodes. In-phase stimulation (0° phase difference between targeted cortical areas) was applied during the positive cycle, and out-of-phase stimulation (180° phase difference between targeted areas) during the negative cycle. Figure 2 illustrates a sample montage for each stimulation condition.

### 2.5. Task Conditions

#### 2.5.1. In-Phase Stimulation

Participants completed 25 min blocks of a speech entrainment task with 15 min of washout (total session duration = 40 min) while receiving in-phase HD-tACS. Electrodes were placed to maximize current flow to the left IFGpo and pMTG/pSTG regions using a ring montage. In-phase stimulation, applied to two brain regions with a 0° relative phase difference, has been shown to enhance network synchronization and improve performance [40,89].

#### 2.5.2. Anti-Phase Stimulation

The electrode placement and behavioral task were identical to those in the in-phase condition, but the current was applied with a 180° phase difference between the targeted areas to disrupt synchronization and impair performance [53].

#### 2.5.3. Sham Condition

In the sham condition, stimulation mimicked the sensation of active stimulation but lasted only 30 s, with gradual ramping up and down to prevent participants from distinguishing it from the active conditions [53]. This approach minimizes the likelihood that participants will recognize the sham condition [82,90].

### 2.6. Outcome Measures

Three outcome measures were evaluated: (1) number of words produced, (2) number of errors, and (3) entrainment as measured by the distance between the SE model and patient productions. Outcomes were evaluated for each stimulation condition (in-phase, anti-phase, and sham). Transcripts from discourse and speech entrainment tasks were analyzed using the CHAT (Codes for the Human Analysis of Transcripts) format, which integrates with CLAN (Computerized Language Analysis Tools) tools to assess linguistic and discourse structures [91]. Although initially used for child language research, CHAT-CLAN has been adapted for aphasia studies [92]. MOR, FREQ, and SCRIPT commands were used for data analysis. Graduate student clinicians trained in CHAT-CLAN procedures, supervised by certified speech-language pathologists, rated the transcripts. Despite no second ratings, feedback was shared to resolve coding disagreements. The discourse team maintained excellent inter- and intra-rater reliability (0.82–0.98) in previous studies [93].

The *number of words* was counted to reflect total speech output across conditions. Unintelligible words, neologisms, repetitions, and revisions were excluded per the CLAN manual. The *proportion of errors* derived from the CLAN SCRIPT analysis reflects the proportion of errors in each participant’s script production relative to the total number of script words.

To determine the extent to which participants were “entrained” to the audiovisual (AV) model, a dynamic time warping algorithm was used to determine the distance between the productions from the AV model and the participant’s production. Dynamic time warping (DTW) is a method for comparing two temporal sequences that do not align. To determine the distance between the two samples, DTW shifts the time series and maps each element in one series to the closest element in the other, thereby finding the optimal distance between the two. DTW has been used to analyze pathological speech and language, including post-stroke aphasia [94,95]. FAST DTW, a dynamic time warping algorithm [96], was implemented in Python (librosa 0.9.1 library) [97] to determine optimal alignments between model and participant audio samples. A mel-frequency cepstrum (MFC) analysis was conducted to derive a metric for determining the distance between two audio samples. The MFC is composed of mel-frequency cepstral coefficients (MFCCs [98]), a spectral feature used in speech recognition systems. MFCCs are derived by taking the Fourier transform of a signal, mapping the powers of the spectrum onto the mel scale, taking the logs of the powers at each mel frequency, and taking the discrete cosine transform of the list of mel log powers. The amplitudes of the resulting spectrum are the MFCCs. Using the MFCCs, an array of feature vectors can be extracted and compared with a dynamic time warping algorithm to calculate a normalized distance between the two feature vectors. The distance measure is the sum of the corrections needed to “warp” the participant onto the model. Distances were calculated for each of the three productions of the final script of the stimulation period. Parameters are available at https://github.com/Kylerpspell/MFCC_DTW (accessed on 18 March 2026). Across all samples, the distance metric was normalized to address the discrepancy in the number of feature vectors, due to approximately 2% variance (+/− 200 vectors, averaging 10,500) across audio samples. It is important to note that the values calculated from the MFCC vectors are absolute distance measures, not explicitly relative. For example, two trials may have very similar distances but represent distinct speaker productions. Higher values indicate a greater distance between the speaker and the model, while lower values indicate a smaller distance.

### 2.7. Statistical Analyses

All statistical analyses were conducted using R (4.5.2) and R Studio (Posit Software, PBC; Version 2026.01.1 + 403). Descriptive statistics (means and standard deviations) for all behavioral outcomes were reported across the three conditions. The Shapiro–Wilk test revealed that the linguistic and temporal-acoustic behavioral variables were not normally distributed (*p* > 0.05) with ties. Therefore, nonparametric methods (Friedman and post hoc Nemenyi tests) were used. A Friedman test was used to assess differences in performance for each HD-tACS condition: (1) in-phase; (2) anti-phase; and (3) sham. To assess the magnitude of the differences, Kendall’s *W* coefficient of concordance was calculated as the effect size.

## 3. Results

### 3.1. Demographics and Clinical Characteristics

Of the 30 participants contacted, 18 enrolled, and 17 completed the study. One participant did not complete all sessions. Twelve individuals were classified as ‘screening failures’ due to not meeting the inclusion criteria or declining participation.

The final analysis included 13 participants, as behavioral data from four participants (T9, T11, T16, T18) were identified as outliers using the interquartile range criterion for each outcome measure, likely due to limited verbal output caused by severe aphasia and motor speech disorders like apraxia, and were excluded from further analysis.

**Figure 3:**
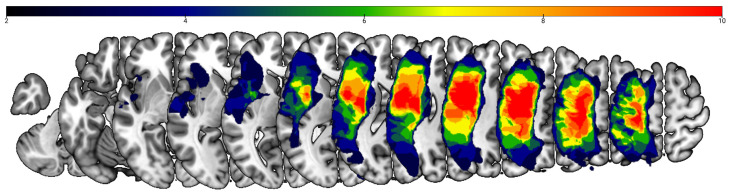
Lesion overlay map (n = 13). The color scale indicates the number of participants with damage at a given cortical location. The upper boundary of the color scale represents the area of highest lesion overlap.

The cohort of 13 participants included 5 women (38%). All thirteen individuals had had a left-hemisphere stroke and were at least one year post-stroke (mean months post-onset = 103.15, SD = 74.71, range = 36–298). The average age was 64.31 years (SD = 10.27, range = 48–77), with an average education level of 15.54 years (SD = 2.47, range = 12–20). Participants identified as White (n = 10) or Black/African American (n = 3). All had nonfluent aphasia (mean AQ = 66.58, range = 30.8–82.0). Demographic and lesion data are available in Table 1 with a lesion overlap map in Figure 3.

### 3.2. Blinding and Discomfort Ratings

Upon completion of each behavioral session, participants were asked whether they believed they received active stimulation (in-phase or anti-phase) or the sham condition. The SLP administering the behavioral sessions also completed the query. Patient reports and actual stimulation conditions were not significantly different from chance (*p* < 0.48). SLP reports were also not significantly different from chance (*p* < 0.92). This suggests that the participants and administrator were effectively blinded to stimulation conditions.

Patients reported 0 out of 10 pain and/or discomfort during stimulation conditions (mean = 0; SD = 0) and 0–1 pain and/or discomfort (mean = 0.08; SD = 0.27) during active stimulation conditions. Statistical analyses revealed that discomfort ratings were comparable between the sham and active stimulation conditions (Mann–Whitney U; *p* = 0.23), indicating that patients did not report a significant difference in discomfort level across conditions.

### 3.3. Outcome Measures

#### 3.3.1. Number of Words

**Figure 4:**
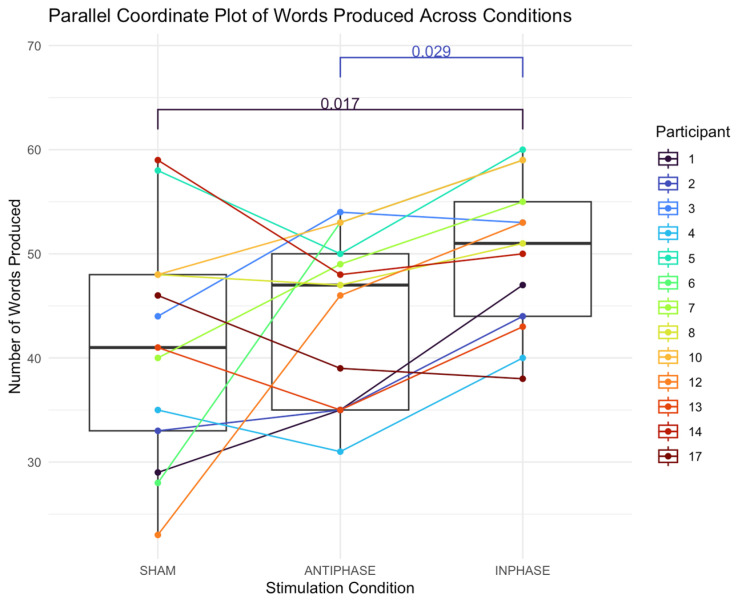
Parallel coordinate plot illustrating individual participants’ number of words produced across the three stimulation conditions (sham, anti-phase, and in-phase). Lines represent within-participant trajectories. Each line shows a participant’s score under the three stimulation conditions; boxes summarize medians and IQRs.

The median number of words in the sham condition was 41 (IQR: 15; 38.5–53.0). The median number of words in the anti-phase condition was 47 (IQR: 15; 44–55). The median number of words in the in-phase condition was 51 (IQR: 11; 44–55). A Friedman test revealed a significant main effect of condition on the number of words produced: *χ*^2^ (2) = 9.38, *p* = 0.009 (Figure 4). The effect size, calculated using Kendall’s *W*, was 0.36, indicating a *moderate* effect (Cohen’s guidelines for W [99]: 0.1–<0.3 (small), 0.3–<0.5 (moderate), and ≥0.5 (large)). Post hoc analysis was performed using Nemenyi’s test with a multiple-comparison adjustment to determine which conditions differed significantly. The results revealed significant differences between in-phase stimulation and sham conditions (*p* = 0.017) and anti-phase stimulation and in-phase conditions (*p* = 0.029).

#### 3.3.2. Number of Errors

**Figure 5:**
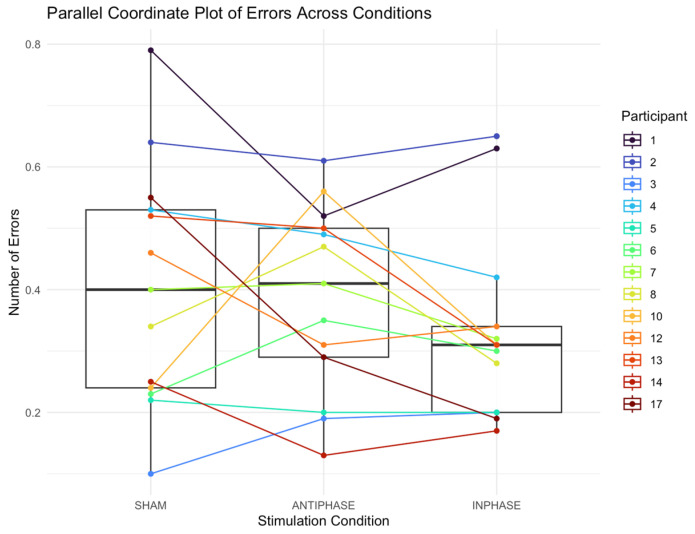
Parallel coordinate plot showing individual participants’ errors produced across sham, anti-phase, and in-phase stimulation. Each line represents a participant’s score under the three stimulation conditions; boxes summarize medians and IQRs.

The median number of errors (reported in proportion of errors produced across the script) in the sham condition was 0.40 (IQR: 0.29; 0.24–0.53). The median number of errors in the anti-phase condition was 0.41 (IQR: 0.21; 0.29–0.50). The median number of errors in the in-phase condition was 0.31 (IQR: 0.14; 0.20–0.34). A Friedman test found no significant effect on the number of errors produced across conditions: *χ*^2^ (2) = 2.24, *p* = 0.327 (Figure 5).

#### 3.3.3. Entrainment

**Figure 6:**
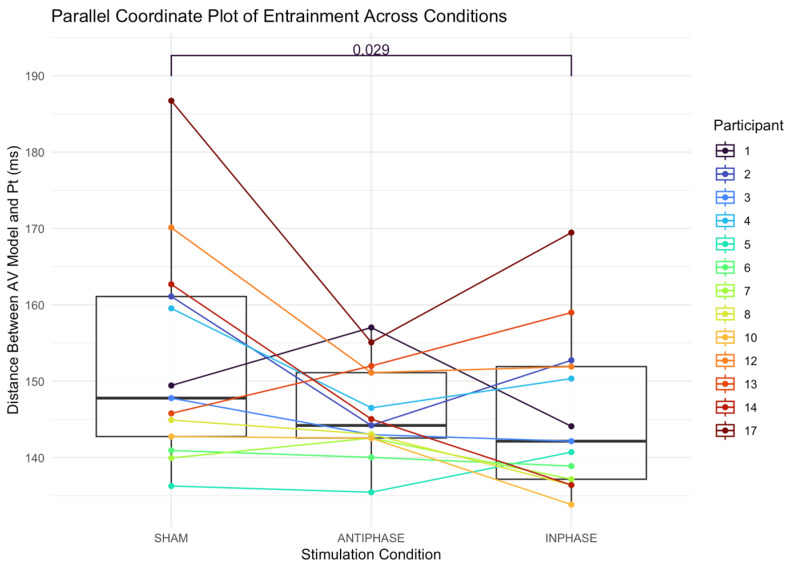
Parallel coordinate plot illustrating individual participants’ entrainment (as measured by distance between participant production and the audiovisual speech entrainment model) across the three stimulation conditions (sham, anti-phase, and in-phase). Lines represent within-participant trajectories. Each line shows a participant’s score under the three stimulation conditions; boxes summarize medians and IQRs. Pt = participant; AV = audiovisual.

The median distance between the audiovisual model and the participant’s production in the sham condition was 147.79 ms (IQR: 18.33; 142.76–161.09). The median distance in the anti-phase condition was 144.21 ms (IQR: 8.56; 142.56–151.12). The median distance in the in-phase condition was 142.14 ms (IQR: 14.75; 137.16–151.91). A Friedman test revealed a significant main effect of distance between the speech entrainment model and participant productions across stimulation conditions: *χ*^2^(2) = 7.54, *p* = 0.023 (Figure 6). The effect size, calculated using Kendall’s *W*, was 0.29, indicating a small effect (Cohen’s guidelines for W [99]: 0.1–< 0.3 (small), 0.3–< 0.5 (moderate), and ≥0.5 (large). Post hoc analysis was performed using Nemenyi’s test with a multiple-comparison adjustment to determine which conditions differed significantly. The results revealed significant differences between in-phase stimulation and sham conditions (*p* = 0.029), with participants being more entrained (i.e., better temporally aligned, as measured by a smaller distance between the model and the patient’s production).

## 4. Discussion

The primary goal of this investigation was to conduct a proof-of-concept study to evaluate the potential of HD-tACS at 7 Hz to enhance speech output in individuals with chronic, nonfluent aphasia during a speech entrainment task. Group-level analyses for two of the three outcome variables suggest that in-phase tACS stimulation improves speech output. A greater number of words were produced during the in-phase condition, and participants demonstrated greater entrainment to the model during the in-phase condition. It is also encouraging to note that, although not statistically significant at the group level, participants tended to produce fewer errors during the in-phase condition.

The results suggest that for at least some participants, the in-phase stimulation elicited more speech and better entrainment. For others, however, behavioral performance is best during anti-phase stimulation as compared to sham and in-phase stimulation. For example, participants 3 and 17 produce more words in the anti-phase condition compared to the in-phase condition. With respect to the temporal data, participants 2, 4, 5, 13, and 17 demonstrate better entrainment during the anti-phase condition as compared to the in-phase condition. It is worth noting that participant 17 also had the second-largest lesion size in the cohort (234,736 mm^3^). Some patients also demonstrate improved performance with both types of stimulation compared to the sham condition, to varying degrees. Although cortical tracking or electrophysiological methods were not explicitly tested in the current study, it seems reasonable to speculate that, for those who performed better in the anti-phase condition than in the sham condition, stimulation recruited at least some residual cortical regions or elicited some degree of coherence in the left hemisphere. It may also be true for these participants that the benefit of anti-phase stimulation lies not so much in the synchronization as in the presence of local stimulation.

The findings are important as they suggest tACS may be a novel neuromodulatory technique to enhance outcomes as an adjuvant to behavioral therapies. The task that was included in the current study is also important to consider. The use of rhythm in clinical approaches to nonfluent aphasia is not new and has been used extensively for decades to improve language fluency. The nature of speech entrainment, particularly as a rehabilitation paradigm for nonfluent aphasia, has been investigated by Fridriksson and colleagues over the last decade [14,15,25,26]. The underlying nature of entrainment and the rhythmicity of human speech are likely important factors in the entrainment observed in the current study. Speech entrainment not only capitalizes on the notion of errorless learning but is also thought to rely on rhythm as an active therapeutic ingredient [100,101]. In this study, we capitalized on the rhythmic nature of the speech entrainment paradigm, pairing it with a modulatory rhythm source: HD tACS. Further, tACS was delivered at 7 Hz, a frequency thought to facilitate speech processing [102].

From a clinical perspective, implementing speech entrainment as a behavioral intervention is feasible, and scripts can be modified to target functional or personally salient topics. Clinicians may also adapt scripts to address specific linguistic impairments (e.g., targeting agrammatism by modifying syntactic structure). Regarding noninvasive brain stimulation, there is growing evidence that it is an effective adjuvant to aphasia therapy [103]. What is not clear, however, is how and when noninvasive brain stimulation techniques will be successfully integrated into clinical practice. Speech-language pathologists, the primary rehabilitation professionals treating speech and language following a stroke, report clinical interest in integrating neuromodulation in the clinical setting; however, several barriers remain [104,105].

This study presents a novel application of alternating electrical current stimulation to improve speech production during speech entrainment in individuals with nonfluent aphasia. The observed trends during in-phase stimulation compared to sham suggest that HD-tACS may hold promise as a therapeutic tool for improving speech outcomes in stroke survivors with aphasia. The results also underscore the heterogeneity of nonfluent aphasia and the need to better understand the individual mechanisms underlying recovery. Although the current findings are preliminary, they provide a rationale for further exploration of HD-tACS in larger, more targeted studies. Additionally, electrophysiological measures, such as electroencephalography (EEG) or magnetoencephalography (MEG), should be paired with HD-tACS to better understand the underlying neural mechanisms and how tACS modulates them in post-stroke aphasia. It is also worthwhile to consider how the effects of tACS compare to those of other neuromodulatory methods that have demonstrated success in this clinical population (i.e., tDCS).

Several limitations must be acknowledged. This study examined the effects of HD-tACS on a small cohort of participants. Across participants, there is an inherent variability in stroke and aphasia recovery as well as in response to neuromodulation. Moreover, motor speech disorders, such as apraxia of speech and dysarthria, were not considered exclusion criteria in this study, which may have influenced the behavioral outcomes. Future research should aim to standardize inclusion criteria and account for these confounding factors to better isolate the effects of HD-tACS on speech production [41,106]. Additionally, while sham conditions are often considered the gold standard in brain stimulation studies, the perceptual effects of alternating current stimulation can vary across individuals, potentially complicating the interpretation of sham-controlled results. Future studies should consider these factors and explore optimized stimulation parameters, such as amplitude, frequency, and duration, to enhance the efficacy of HD-tACS.

In conclusion, this proof-of-concept study suggests that HD-tACS may enhance speech production in individuals with nonfluent aphasia. The findings offer a promising foundation for future research. Larger, more rigorous studies are needed to fully understand the therapeutic potential of HD-tACS and to optimize its application for post-stroke aphasia rehabilitation. The concept of a “tACS boost” in speech production warrants further investigation, and if validated, could have significant implications for the treatment of chronic aphasia.

## Figures and Tables

**Table 1:** Demographic data for thirteen participants included in the final analysis. MPO = month post onset; WAB-R AQ = Western Aphasia Battery Aphasia Quotient; AOS = Apraxia of speech. Age and education are measured in years. AOS is indicated by presence (+) or absence (–). WAB-R AQ is scored on a scale of 0–100, with lower scores indicating a more severe language deficit. Lesion Volume is reported in mm^3^.

Pt ID	Age	Education	Sex	Race	MPO	WAB-R AQ	AOS	Lesion Volume
**T1**	75	12	Female	White	93	30.8	+	113,410
**T2**	60	16	Male	White	135	52.8	+	243,361
**T3**	65	16	Male	White	75	66.5	–	219,675
**T4**	47	16	Female	White	190	62.5	+	59,149
**T5**	62	12	Male	Black	41	80.8	–	42,579
**T6**	71	16	Male	White	82	63.2	+	210,969
**T7**	63	18	Male	White	153	74.7	–	148,221
**T8**	70	14	Male	White	38	82	–	57,123
**T10**	48	16	Male	Black	68	79.9	+	53,438
**T12**	54	12	Male	Black	63	72.5	–	177,110
**T13**	79	18	Female	White	36	81.6	–	95,419
**T14**	65	20	Male	White	69	78.1	–	20,257
**T17**	77	16	Male	White	298	40.2	–	234,736
**Mean**	64.31	15.54	5 Females	10 white	103.15	66.58	55%	
**SD**	10.27	2.47	-	-	74.71	16.51	–	

## Data Availability

The data that support the findings of this study are available upon on request from the corresponding author, upon reasonable request.

## Abbreviations

The following abbreviations are used in this manuscript:

HD-tACS	High-definition transcranial alternating current stimulation
STG	Superior temporal gyrus
SE	Speech entrainment
IFGpo	Inferior frontal gyrus pars opercularis
pMTG	Posterior middle temporal gyrus
tDCS	Transcranial direct current stimulation
SLP	Speech-language pathologist
MRI	Magnetic resonance imaging
AQ	Aphasia quotient
